# Globalization, Women, and Infectious Diseases^1^

**DOI:** 10.3201/eid1011.040623_11

**Published:** 2004-11

**Authors:** Gillian Howey Kimura, Simon N. Mardel, Paul Courtright, Susan Watts

**Affiliations:** * Department of Health and Human Services, Washington, DC, USA;; † World Health Organization, Geneva, Switzerland;; ‡ Tumaini University, Meshi, Tanzania;; § American University in Cairo, Cairo, Egypt

**Keywords:** infection control, nosocomial, eye disease, trachoma, blind, water, sanitation, schistosomiasis

Globally, women bear the primary responsibilities for childrearing, caregiving, water gathering, cleaning, and other family-related tasks. In developing countries, these activities can expose women disproportionately to infectious disease. Once infected, women often face further disparities in their diagnosis and treatment options.

## Infection Control, Universal Rights

Inadequacies of infection control are exemplified by viral hemorrhagic fever outbreaks. Ebola virus causes severe progressive weakness, often with vomiting, diarrhea, and bleeding. Healthcare workers are charged with managing these patients, which has led to high rates of nosocomial transmission among staff. Because of the high number of female caregivers, women are disproportionately affected when universal precautions are ignored. The immediate instinct is to treat the patient before cleaning vomit or other bodily fluids from the area. A more appropriate response of cleaning and disinfecting, strictly controlling and disposing of sharps, and wearing latex gloves is recommended. The goal should be always to protect staff and caregivers first. Solutions include educating patients and healthcare workers about their rights related to infection control.

## Disparities in Infectious Eye Diseases in Developing Countries

Globally, two blind women exist for every blind man. Age-adjusted odds of blindness in women compared to men worldwide is 1.43. Trachoma is the leading infectious cause of blindness. *Chlamydia trachomatis* is spread by hand-eye contact, flies, and sharing of towels. Sex is linked to higher exposure to *C. trachomatis* and progression to active disease, trichiasis, corneal scarring, and blindness; sex also plays a role during the development of conjunctival scarring. A sex-sensitive approach is necessary for both primary prevention as well as use of eye care services, including early recognition of the need for surgery. A sex-sensitive approach would include interacting with women as primary stakeholders in the process. Women's needs and opinions should be taken into consideration when designing prevention programs, including determining what barriers they may face in changing behaviors, if the program is to be effective. A recommendation was made for primary prevention through the SAFE model: surgery (to correct trichiasis), antimicrobial drugs (to treat and decrease the community pool of *C. trachomatis*), face washing (to interrupt transmission), and environmental improvements (to interrupt transmission).

## Women and Water-related Diseases in Rural Egypt

Women play a major role in the collection, storage, use, and disposal of water. Women's proper use of water is essential to the health of family and community. Diseases associated with lack of safe water or sanitation include diarrheal diseases, schistosomiasis, and trachoma. Schistosomiasis has steadily decreased in Egypt due in part to the National Schistosomiasis Control Program. This program focuses on free diagnosis and treatment, snail control, health education, and linkage to water and sanitation improvement. Experience gained from schistosomiasis prevention efforts may apply to other water-related diseases. Behavior change is a complex process that takes time and is difficult to measure precisely. Safe water sanitation, maintenance, and use can help prevent disease transmission.

